# Fat intake impairs the recovery of endothelial function following mental stress in young healthy adults

**DOI:** 10.3389/fnut.2023.1275708

**Published:** 2023-11-09

**Authors:** Rosalind Baynham, Samuel R. C. Weaver, Catarina Rendeiro, Jet J. C. S. Veldhuijzen van Zanten

**Affiliations:** ^1^School of Sport, Exercise and Rehabilitation Sciences, University of Birmingham, Birmingham, United Kingdom; ^2^Centre for Human Brain Health, University of Birmingham, Birmingham, United Kingdom

**Keywords:** high-fat, mental stress, vascular, endothelial function, flow-mediated dilatation

## Abstract

**Introduction:**

Mental stress has been identified as a trigger of cardiovascular events. A single episode of stress can induce acute impairments in endothelial function in healthy adults. Importantly, during stressful periods, individuals often resort to unhealthy behaviors, such as increased consumption of high-fat foods, which is also known to negatively impact endothelial function. Therefore, this study examined whether consumption of a high-fat meal would further exacerbate the negative effect of mental stress on vascular function.

**Methods:**

In a randomized, counterbalanced, cross- over, postprandial intervention study, 21 healthy males and females ingested a high-fat (56.5 g fat) or a low-fat (11.4 g fat) meal 1.5 h before an 8-min mental stress task (Paced-Auditory-Serial-Addition-Task, PASAT). Plasma triglyceride (TAG) concentration was assessed pre-and post-meal. Forearm blood flow (FBF), blood pressure (BP), and cardiovascular activity were assessed pre-meal at rest and post-meal at rest and during stress. Endothelial function, measured by brachial flow-mediated dilatation (FMD) was assessed pre-meal and 30 and 90 min following mental stress.

**Results:**

Plasma TAG concentration was significantly increased following the high-fat meal compared to the low-fat condition. Mental stress induced similar increases in peripheral vasodilation, BP, and cardiovascular activity, and impaired FMD 30 min post-stress, in both conditions. FMD remained significantly impaired 90 min following stress in the high-fat condition only, suggesting that consumption of fat attenuates the recovery of endothelial function following mental stress.

**Discussion:**

Given the prevalence of fat consumption during stressful periods among young adults, these findings have important implications for dietary choices to protect the vasculature during periods of stress.

## 1. Introduction

Stress is extremely prevalent in today’s society, with 74% of the population stating having felt so stressed they are unable to cope (1). Stress has also been linked with both poor physiological and psychological health (2). For example, epidemiological studies have shown that when a population is hit by stressful events such as earthquakes, war, and even losing key football matches, there is an increased incidence of myocardial infarction (3–5). Laboratory studies have shown that mental stress can induce myocardial ischemia (6), and that laboratory-based stress-induced myocardial ischemia is related to ambulatory ischemia (7). Although, the underlying mechanisms are not yet fully understood, impairments in vascular function have been implicated as a possible mechanism. For example, those who experience mental stress-induced myocardial ischemia also have an attenuated peripheral vasodilatory response during stress (8), as well as increased vascular resistance (9, 10).

It has been well established that mental stress evokes increases in heart rate and blood pressure, driven by activation of the sympathetic nervous system and withdrawal of the parasympathetic nervous system (11). Mental stress also impacts the vasculature, and this sympathetic and parasympathetic activation is associated with a nitric oxide (NO)-mediated increase in peripheral vasodilation during mental stress (as measured by forearm blood flow; FBF) (12, 13). Importantly, stress-induced vasodilation is attenuated in populations at risk of cardiovascular disease (CVD), such as obesity (14). Furthermore, mental stress can trigger a transient, but clinically significant, decline in endothelial function (as measured by brachial flow-mediated dilatation; FMD) from 15 to 90 min following stress in young healthy adults (15, 16). Potential mechanisms have been suggested to involve stress-induced increases in cortico-releasing hormone (CRH), cortisol, and pro-inflammatory cytokines (15), as well as up-regulation of oxidative stress (17); all of which can attenuate NO-production and result in endothelial dysfunction (18).

Stress can also influence physical health indirectly through changes in behavior (19) and adoption of maladaptive coping mechanisms (2). Importantly, stress can impact eating patterns, with studies reporting 42% of individuals to consume more, and more often unhealthy foods (i.e., high-fat and sugar) during stressful periods (20–22). For instance, young adults are more likely to choose foods with higher levels of fat following stress compared to a no stress condition (23). Crucially, fat intake can negatively impact the vasculature: brachial FMD is reported to be impaired for 8 h following consumption of a high-fat meal in healthy and clinical populations (24–26). Hypertriglyceridemia and hyperglycemia following fat consumption (27, 28) have been shown to stimulate the vasoconstrictor endothelin-1 (ET-1), reactive oxygen species (ROS) and inflammatory markers (27, 29), which subsequently reduce endothelium-derived NO (30). Reduced NO production is implicated as a major mechanism driving fat-induced endothelial dysfunction. Furthermore, impaired resting endothelial function has been associated with poorer vascular responses to stress (31). As such, it is likely that increased fat intake during stress further aggravates the effect of stress on the vasculature. Given the high prevalence of fat consumption during stressful periods it is important to determine the full impact of such interactions on human vascular health.

To our knowledge, only one study has previously attempted to address this question using a model of repeated stress, but possibly due to a relatively low number of participants (*N* = 10) and timing of FMD measurements, did not show effects of stress and fat separately on FMD or an interaction between stress and fat (32). The current study aimed to investigate the effect of a high-fat meal on peripheral (FBF) blood flow as well as endothelial function (FMD) in healthy adults in the context of a mental stress challenge. We hypothesized that a high-fat meal will impair peripheral blood flow during stress and exacerbate mental stress-induced endothelial dysfunction, compared to a low-fat meal.

## 2. Materials and methods

### 2.1. Participants

Twenty-one participants (11 male, 10 female) were recruited via email and poster advertisements. Females were tested during the same phase of the menstrual cycle (early follicular, days 1–5 of menstruation) to control for the influence of menstrual hormones. Participants were between 18 and 45 years old. Exclusion criteria were: (i) smokers, (ii) consumption of >21 units alcohol per week, (iii) acute illness/infection, (iv) history of cardiovascular, respiratory, metabolic, liver, inflammatory diseases, or blood-clotting disorders, (v) allergies or food intolerances, (vi) weight reducing dietary regiment or dietary supplements, and (vii) long-term medication or antibiotics in the previous 3 months. Participants were awarded course credit marks when applicable. Ethical approval was obtained from the University of Birmingham Science, Technology, Engineering and Mathematics ethics committee (ERN17_1755D), and all participants gave written informed consent prior to participation in the study.

### 2.2. Habitual dietary intake

Habitual dietary intake was assessed using the validated European Prospective Investigation into Diet and Cancer (EPIC) Norfolk Food Frequency Questionnaire (FFQ) (33). Participants recalled their usual dietary intake over the previous 12 months, with 131 different food items, on a 9-point scale (never or less than once per month, 1–3 per month, once a week, 2–4 per week, 5–6 per week, once a day, 2–3 per day, 4–5 per day, and 6+ per day). The FFQ EPIC Tool for Analysis (FETA) was used to calculate nutrient data (34). The following nutrients are reported in this study: energy (kcal), fat (g), saturated fat (g), carbohydrate (g), sugars (g), fiber (g), protein (g) and portions of fruit and vegetables (calculated as 1 portion corresponding to 80 g), to give a general view of habitual dietary intake.

### 2.3. Study design

**Figure 1:**
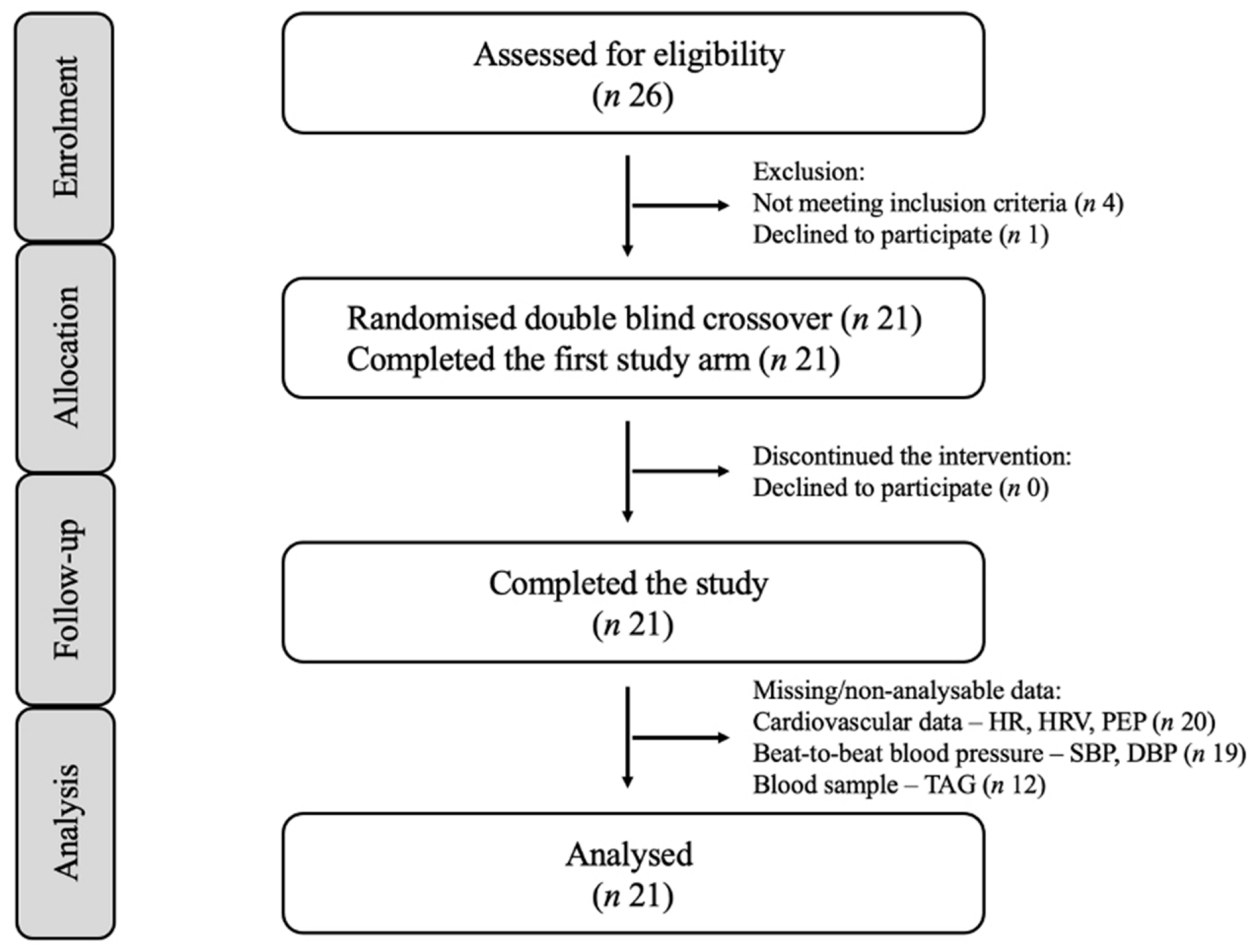
Consolidated standards of reporting trials (CONSORT) flow diagram for postprandial intervention study.

**Figure 2:**
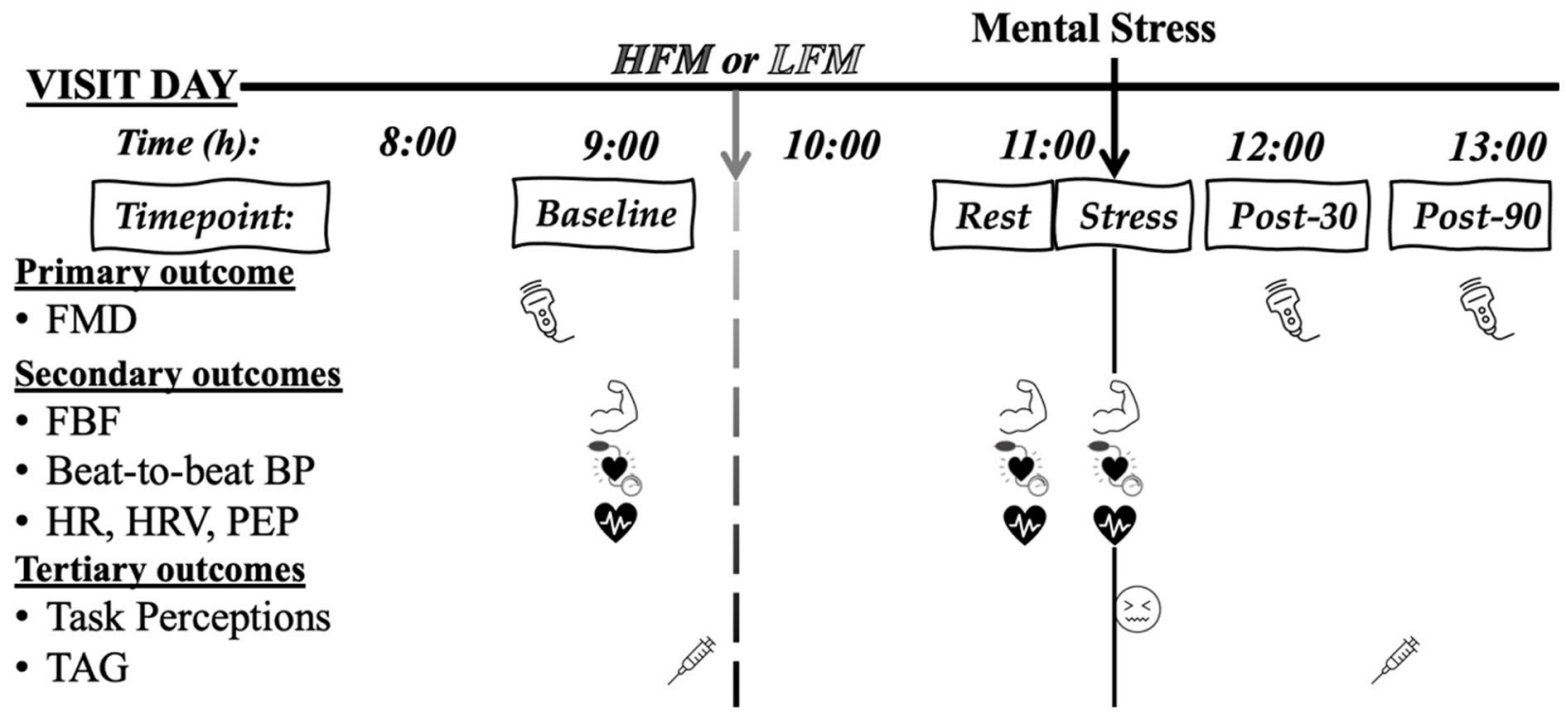
Experimental study design.

The study design was a randomized, counterbalanced, cross-over, postprandial intervention study (Figure 1). Participants visited the laboratory twice, at least a week apart for males and approximately 1 month apart for females. Participants were asked to refrain from food for 12 h and from alcohol, vigorous exercise, and caffeine 24 h before each testing session. Each session commenced at approximately 8 AM, and firstly, compliance with pre-visit requirements were checked. Participants were then instrumented with equipment to measure cardiovascular activity. Following this, participants rested in a supine position for 20 min before pre-intervention (Baseline) measurements were taken: (i) brachial FMD, (ii) FBF, (iii) cardiovascular activity (beat-to-beat blood pressure [BP], heart rate [HR], heart rate variability [HRV] and pre-ejection period [PEP]); (iv) blood sample (to measure plasma triglycerides [TAG] concentration). Following these assessments, participants consumed either a high-fat meal (HFM) or a low-fat meal (LFM). Participants then rested for 1.5 h during which they completed lifestyle questionnaires (only habitual dietary data reported, session 1) and had the option to complete their own work or watch a nature documentary. Subsequently, FBF and cardiovascular activity were measured during an 8-min rest (Rest) and during an 8-min mental stress – Paced-Auditory-Serial-Addition-Task (PASAT) (Stress). During each 8-min assessment, FBF was measured during minutes 2, 4, 6, and 8. BP, HR, PEP and HRV were analysed for these minutes. Brachial FMD was measured 30 and 90 min following stress. A second blood sample to measure TAG concentration was taken 45 min following stress. A trained researcher carried out all measurements and analyses. Both sessions lasted 5 h and participants were debriefed following completion of both visits (Figure 2).

### 2.4. High- and low-fat interventions

The HFM and LFM were prepared just before consumption, and all fresh ingredients were bought within 24 h of each testing session. The meals were calorie matched, with the HFM containing 56.5 g fat and the LFM containing 11.4 g fat (24) (Table 1). All other nutrients were as closely matched as possible, except for carbohydrate as a higher level of carbohydrate in the LFM was necessary to match caloric intake. Participants were asked to consume the meal within 20 min. Seven participants were not able to finish the low-fat meal and 2 participants were not able to finish the high-fat meal, but no adverse side effects were reported. Whilst it was impossible to blind experimenters and volunteers to the interventions during the visits, these were blinded during all data analyses.

**Table 1:** Nutrient composition of the high-fat and low-fat meals.

Meal type	High-fat meal^1^	Low-fat meal^2^
*Nutrient composition:*		
Energy (Kcal)	891.0	886.0
**Fat (g)**	**56.5**	**11.4**
**Saturated fat (g)**	**35.1**	**5.6**
Carbohydrate (g)	65.0	160.1
Sugars (g)	20.2	19.4
Fiber (g)	2.4	5.9
Protein (g)	29.9	33.3
Salt (g)	2.0	2.5

^1^This meal consists of 2 butter croissants with 10 g salted butter, 1.5 slices of cheese and 250 ml whole milk. ^2^This meal consists of 4 slices of white bread with 30 g Philadelphia light spread, 90 g so-organic cornflakes and 250 ml semi-skimmed milk.

### 2.5. Blood sampling and plasma triglycerides analysis

Blood samples were collected to assess fasting and post-meal plasma TAG concentration. Blood samples were collected in EDTA-coated 10 ml tubes by a trained phlebotomist, from the antecubital vein of the arm. The samples were immediately centrifuged at 5000 rpm for 10 min at 4°C to separate the plasma. 1,000 μl of plasma was pipetted into 1 aliquot for TAG analysis, and stored at −80°C for future assessment. Plasma was later analyzed using commercially available kits for TAG concentration (Triglyceride Kit, Randox, London, United Kingdom), using an automated photometric clinical chemistry analyzer RC Daytona+ (Randox). Samples were analyzed in duplicates, with a coefficient of variation (CV) of 0.44%.

### 2.6. Mental stress task

The mental stress task used was the 8-min PASAT, shown to have good test–retest reliability and to induce a physiological response (12, 35, 36). The PASAT requires participants to add two sequentially presented single-digit numbers (1–9), adding the number presented to the previous number they heard. The delivery of the numbers became quicker, with time intervals reducing every 2 min; from a 2.8 s interval to 2.4 s, 2.0 s, and finally 1.6 s. Participants were filmed and asked to watch themselves on the screen, which they were told would be evaluated by 2 independent body language assessors. An experimenter marked the participants’ responses, whilst sounding a loud aversive buzzer-noise at standard intervals once every 10 answers: either following an incorrect response or at the end of the 10-number block. The participants were told they were in direct competition with other participants and lost points for each incorrect answer. These elements of social evaluation, punishment, and competition have been used previously (37) and have been shown to enhance the provocativeness of the task (38). Immediately following the PASAT, an experimenter asked the participant to verbally rate how difficult, stressful, competitive, and enjoyable they found the task, and to what extent they were trying to perform well, scored on a 7-point scale ranging from 0 ‘not at all’ to 6 ‘extremely’. Following completion of both visits, participants were informed about the deception of the task.

### 2.7. Cardiovascular activity

#### 2.7.1. Impedance cardiography

The Ambulatory Monitoring System, VU-AMS5s (TD-FPP, Vrije Universiteit, Amsterdam, Netherlands) was used to continuously record an electrocardiogram (ECG) and impedance cardiogram (ICG) to measure heart rate (HR, bpm), heart rate variability (HRV, ms – a measure of parasympathetic activity) and pre-ejection period (PEP, ms – a measure of sympathetic activity) in line with published guidelines (39, 40). The VuAMS5fs was connected to 7 Ag/AgCl spot electrodes (Invisatrace, ConMed Corpo- ration; Largo, FL, USA). ECG electrodes were placed below the right clavicle, between the lower 2 ribs on the right side, and at the apex of the heart on the left lateral margin of the chest. ICG electrodes were placed at the top end of the sternum at the suprasternal notch and at the bottom of the sternum at the xiphoid process, and on the spine, 3 cm above and 3 cm below the upper and lower electrodes, respectively. Analyses were undertaken offline using VU-DAMS software with manual inspection and correction of ECG and averaged ICG data, used to derive HR, HRV, and PEP, averaged for each minute of assessment. HRV was calculated from beat-to-beat ECG data as the square root of the mean of the sum of the squared successive differences in cardiac inter-beat intervals. PEP was defined as the time between Q-wave onset and commencement of systole (39, 40).

#### 2.7.2. Beat-to-beat blood pressure

Beat-to-beat arterial BP was measured using a Finometer (Finapres Medical Systems; Amsterdam, Netherlands), with a cuff around the intermediate phalanx of the middle finger. Continuous data was recorded via a Power1401 (CED, Cambridge, UK) connected to a computer programmed in Spike2. Data was analyzed for the same minutes as FBF was recorded and averaged for each minute of assessment. Analyses were undertaken offline whereby each file was visually inspected, and systolic blood pressure (SBP), diastolic blood pressure (DBP) and mean arterial pressure (MAP) were obtained.

### 2.8. Forearm blood flow

Forearm blood flow was measured using venous occlusion plethysmography. A mercury-in-silastic strain gauge was connected to a plethysmograph (ECG, Hokanson; Jacksonville, WA, USA), producing an output voltage with frequency 0–25 Hz. The plethysmograph signal was digitized at 100 Hz with 16-bit resolution, via a Power1401 (CED) connected to a computer programmed in Spike2, as previously described by Paine et al. (12). One congestion cuff was placed around the wrist (TMC7, Hokanson), and inflated for 1 min to supra-systolic blood pressure (>220 mmHg). Another congestion cuff was placed around the brachial region of the upper arm (SC12, Hokanson), and inflated for 5 s to above venous pressure (40 mmHg), every 15 s providing 3 blood flow measurements each minute. Blood flow analysis and calibration were undertaken offline using Spike2 (CED). Each increase in limb circumference is identified as a slope, which were averaged to yield a mean blood flow per minute (12). Forearm vascular conductance (FVC) was calculated by dividing FBF by MAP per minute of assessment.

### 2.9. Flow-mediated dilatation

Flow-mediated dilatation was used to assess endothelial function of the brachial artery. A 15–4 Mhz (15 L4 Smart MarK™; Terason, MA, USA) transducer was attached to a Terason Duplex Doppler System (Usmart 3,300 NexGen Ultrasound; Terason). This has a wall-tracking and automatic edge-detection software (Cardiovascular Suite, Quipu; Pisa, Italy), which allows for continuous measurement of diameter and blood velocity throughout the FMD assessment. Following 20 min of supine rest, the brachial artery was imaged longitudinally, 5–10 cm proximal to the antecubital fossa. A brachial cuff was placed around the forearm and, following a 1-min baseline, this was inflated to 220 mmHg for 5 min, to cause ischemia. Subsequently, the rapid cuff deflation caused reactive hyperemia, and the image was recorded continuously for 5 min post-pressure release. This is in accordance with established guidelines (41). All measurements were undertaken by the same trained researcher, who demonstrates sufficient reproducibility in brachial FMD measurements (coefficient of variation: intra-day 5.49%, inter-day 10.87%). All file images were analyzed by the same trained researcher, blinded to condition and measurement details. Peak diameter was defined as the largest diameter obtained after occlusion is released. The FMD response was calculated as the relative diastolic diameter change between baseline and peak diameter. Resting arterial diameter was also estimated based on a time-average across the first minute of recording.

### 2.10. Statistical analysis

All statistical analyses were conducted using IBM SPSS software (version 25). The cardiovascular and FBF measurements during pre-intervention baseline, rest, and stress were averaged separately to provide a mean pre-intervention baseline, rest, and stress value for each outcome. Pre-intervention baseline measures (FMD, FBF, HR, SBP, DBP, TAG), task perceptions and PASAT scores were compared using a 2 condition (HFM, LFM) repeated measures analysis of variance (ANOVA). Plasma TAG concentration was analyzed using a 2 condition (HFM, LFM) by 2 time (baseline, 2 h post-meal) repeated measures ANOVA. Subsequently, a series of 2 condition (HFM, LFM) by 3 time (baseline, rest, stress) repeated measures ANOVAs were conducted to analyze the cardiovascular and FBF variables. FMD (including resting arterial diameter) was analyzed using a 2 condition (HFM, LFM) by 3 time (baseline, post-30, post-90) repeated measures ANOVA. Where appropriate, pairwise comparisons using Bonferroni correction were conducted to investigate significant effects in more detail. All analyses were also conducted with sex as a between-subject variable. All values reported in text, tables, and graphs are mean ± SD. Occasional missing data are reflected in the reported ‘*n*’ values, and include n-1 due to VU-AMS malfunction, n-2 due to Finapres malfunction and n-9 due to participants not willing to have a blood sample or missed sample time-points. Seven participants did not finish the meal. All statistical tests were repeated excluding these 7 participants. The results were broadly similar to the analyses with the full sample; therefore, it was decided to include all participants to maximize power. For all analyses, significance was set at *α* < 0.05.

## 3. Results

### 3.1. Participant characteristics

Participant characteristics are presented in Table 2. Participants were aged 20–30 years old, with a healthy body mass index (BMI) and identified as white European ethnicity (*n* = 19) or Asian ethnicity (*n* = 2). Pre-intervention baseline FMD, FBF, HR, BP, and TAG concentration were similar in both conditions (*n* = 21, Table 2).

**Table 2:** Mean ± SD participant pre-intervention baseline characteristics in the high-fat meal and low-fat meal condition.

Participant characteristics	High-fat meal	Low-fat meal	*p-*value
*N*	21 (M:11, F:10)		/
Age (years)	22.1 ± 2.7		/
BMI (kg/m^2^)	23.62 ± 3.1		/
FMD (%)	5.62 ± 1.33	5.50 ± 1.32	0.474
FBF (mm/100 ml/min)	2.47 ± 0.98	2.21 ± 0.60	0.240
HR (bpm)	59.11 ± 8.70	59.26 ± 7.71	0.736
SBP (mmHg)	123.11 ± 22.23	118.82 ± 15.68	0.487
DBP (mmHg)	56.03 ± 12.49	52.05 ± 9.02	0.234
TAG (mmol/l)	0.78 ± 0.30	0.79 ± 0.28	0.956

N, number, BMI: body mass index; FMD, flow-mediated dilatation; FBF, forearm blood flow; HR, heart rate; SBP, systolic blood pressure; DBP, diastolic blood pressure; TAG, triglycerides.

### 3.2. Habitual dietary intake

Table 3 displays participant’s estimated daily intake of key nutrients and the percentage of participants exceeding or not meeting daily recommendations, as suggested by the National Health Service (NHS). The average daily intake of fat was 59.42 ± 18.85 g (23.8% of participants exceeding the recommended daily intake) and saturated fat was 21.30 ± 6.53 g (19.0% of participants exceeding the recommended daily intake). Fat and saturated fat consumption was similar between males and females. The average intake of fruit and vegetables was 5.71 ± 3.32 portions per day, with females consuming almost 1 extra portion per day. However, 38.1% of participants did not meet the daily recommendations of 5 portions of fruit and vegetables per day. 100% of participants did not meet the suggested recommendations for fiber intake and exceeded the recommendations for sugar intake (Table 3).

**Table 3:** Mean ± SD estimated daily intake of key nutrients.

Nutrients	Sample average	% of participants over/under recommended daily intake
Energy (Kcal)	1576.48 ± 418.93	N/A
Fat (g)	59.42 ± 18.85	23.8% over
Saturated fat (g)	21.30 ± 6.53	19.0% over
Carbohydrate (g)	185.50 ± 57.45	N/A
Sugars (g)	87.27 ± 42.51	100% over
Fiber (g)	14.09 ± 5.72	100% under
Protein (g)	74.34 ± 25.07	N/A
Portions of fruit and vegetables*	5.71 ± 3.32	38.1% under

Recommendations – fat: <70 g/day, saturated fat: <30 g/day (male)/<20 g/day (female), sugar: <30 g/day, fiber: >30 g/day, fruit and vegetables: >5 portions/day. *1 portion = 80 g (NHS guidelines).

### 3.3. Plasma TAG

**Figure 3:**
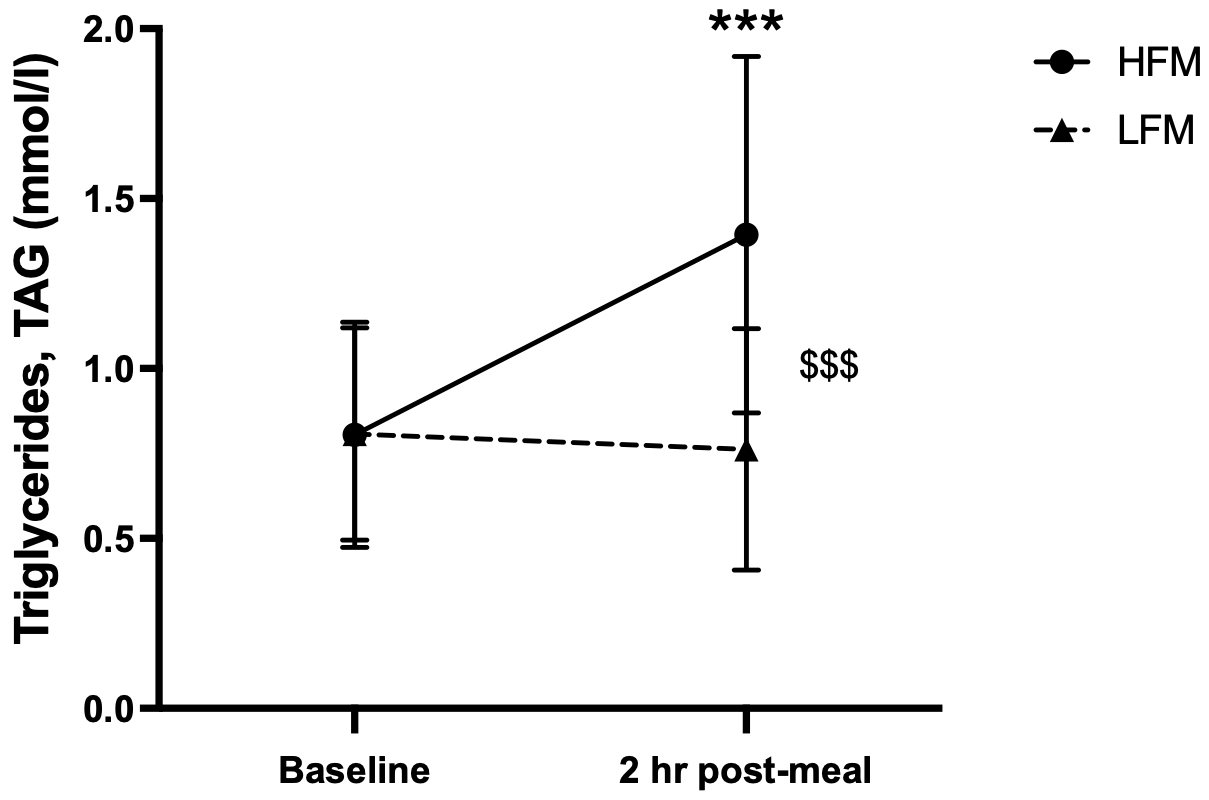
Time course of TAG concentration at baseline and 2 h post-meal. *n* = 12. Data are presented as Mean ± SD. * Significantly different compared to baseline in high-fat meal condition, ^$^ significantly different between conditions, ***/^$$$^*p* < 0.001. HFM, high-fat meal; LFM, low-fat meal.

A 2 condition (HFM, LFM) × 2 time (baseline, 2 h post-meal) ANOVA revealed an overall time effect (*n* = 12, *p* < 0.001), condition effect (*n* = 12, *p* < 0.001) and a condition × time interaction effect (*n* = 12, *p* < 0.001) for TAG concentration (Figure 3). *Post hoc* analyses revealed that TAG concentration was significantly higher after the high-fat meal compared to the low-fat meal (*p* < 0.001), and significantly higher 2 h post-meal compared to pre-intervention baseline (*p* < 0.001). Further exploration of the interaction effect revealed that there was no significant difference in TAG concentration between conditions at pre-intervention baseline (*p* = 0.956), but TAG concentration was significantly higher following the high-fat meal compared to the low-fat meal at 2 h post-meal (*p* < 0.001).

### 3.4. Mental stress task ratings

Separate two condition (HFM, LFM) ANOVAs revealed no significant difference in task performance (PASAT score) or task perceptions between high-fat and low-fat conditions (*n* = 21). Participants perceived the task as similarly difficult, stressful, competitive, enjoyable, and tried to perform well to the same extent after both high-fat and low-fat meals (*n* = 21, Table 4).

**Table 4:** Mean ± SD task performance (PASAT) and ratings (*n* = 21).

Task ratings	High-fat meal	Low-fat meal	*p-*value
PASAT score	141 ± 34	138 ± 35	0.544
Perceived difficulty	4.81 ± 0.60	4.71 ± 0.72	0.576
Perceived stressfulness	4.90 ± 0.94	4.66 ± 0.73	0.204
Perceived competitiveness	4.33 ± 1.20	3.86 ± 1.35	0.135
Perceived enjoyment	1.95 ± 1.16	1.48 ± 1.08	0.125
Perception of trying to perform well	5.00 ± 0.89	5.14 ± 0.96	0.419

Maximum score for PASAT is 228, task ratings are scored from 0 to 6. PASAT, paced-auditory-serial-addition-task.

### 3.5. Cardiovascular activity

**Figure 4:**
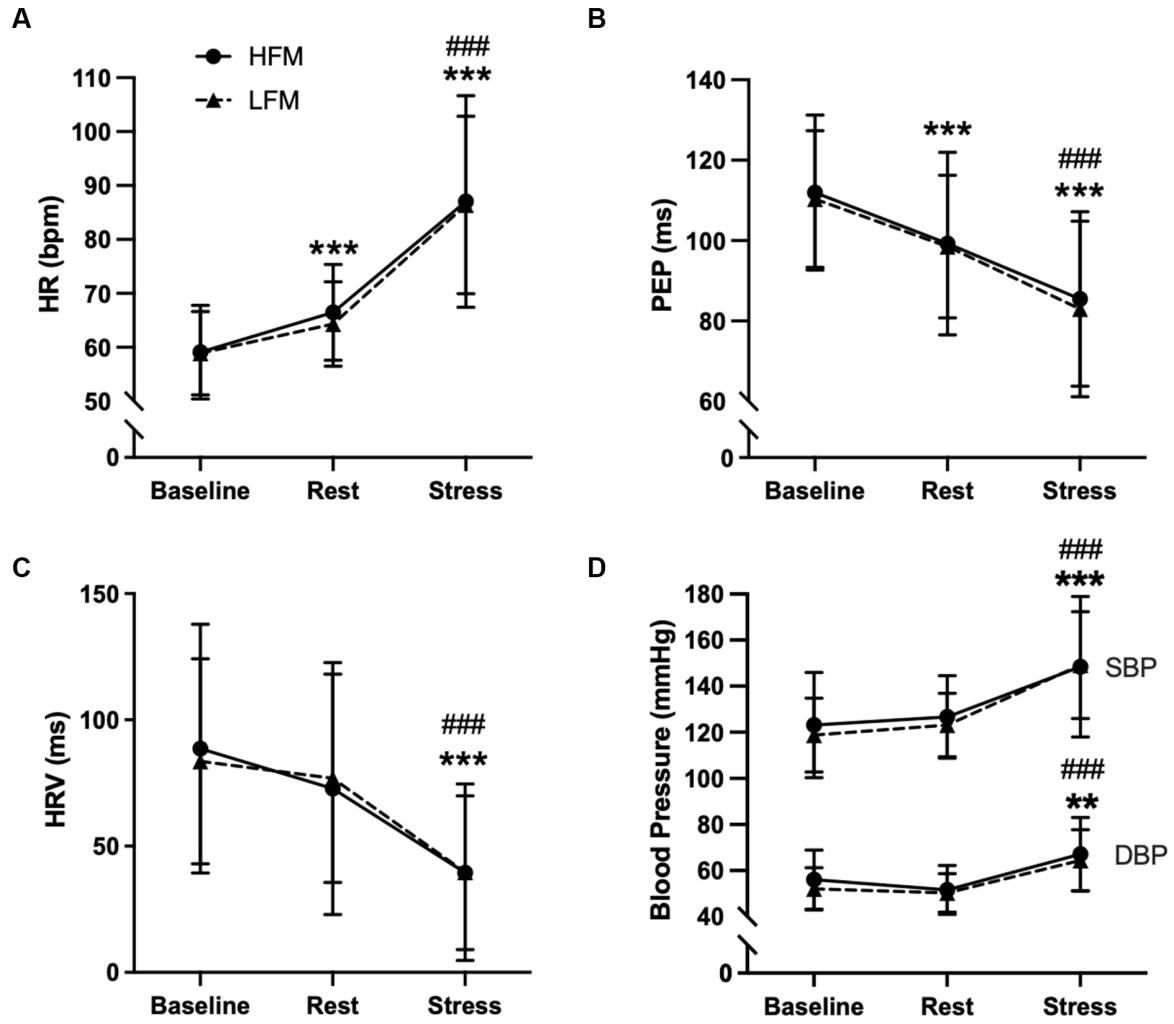
Time course of cardiovascular activity [HR **(A)**, PEP **(B)**, HRV **(C)**, BP **(D)** during baseline, rest and stress following either a high-fat or low-fat meal]. *n*=20 **(A–C)**/*n* = 19 **(D)**. Data are presented as Mean ± SD. * Significantly different from baseline, ^#^ significantly different from rest, ***/^###^*p* < 0.001, **p < 0.01. HR, heart rate; HRV, heart rate variability; PEP, pre-ejection period; SBP, systolic blood pressure; DBP, diastolic blood pressure; HFM, high-fat meal; LFM, low-fat meal.

Separate 2 condition (HFM, LFM) × 3 time (baseline, rest, stress) ANOVAs revealed an overall time effect for HR (*n* = 20, *p* < 0.001), PEP (*n* = 20, *p* < 0.001), HRV (*n* = 20, *p* < 0.001), SBP (*n* = 19, *p* < 0.001) and DBP (*n* = 19, *p* < 0.001) (Figure 4). *Post hoc* analyses revealed that HR was significantly higher during rest compared to baseline (*p* < 0.001) and increased further during stress (*p* < 0.001). HRV was significantly lower during stress compared to baseline and rest (*p*’s < 0.001). Compared to baseline, PEP was significantly lower during rest (p < 0.001), with a further decrease during stress (*p* < 0.001). Both SBP and DBP were significantly higher during stress compared to both baseline (SBP: *p* < 0.001, DBP: *p* = 0.002) and rest (*p*’s < 0.001) and no significant differences were found between pre-intervention baseline and rest (SBP: *p* = 0.492, DBP: *p* = 0.152). There were no significant condition or condition × time interaction effects for HR (condition: *p* = 0.301, interaction: *p* = 0.562), HRV (condition: *p* = 0.773, interaction: *p* = 0.913), PEP (condition: *p* = 0.854, interaction: *p* = 0.608), SBP (condition: *p* = 0.463, interaction *p* = 0.882) or DBP (condition: *p* = 0.269, interaction: *p* = 0.620).

### 3.6. Forearm blood flow during acute mental stress

**Figure 5:**
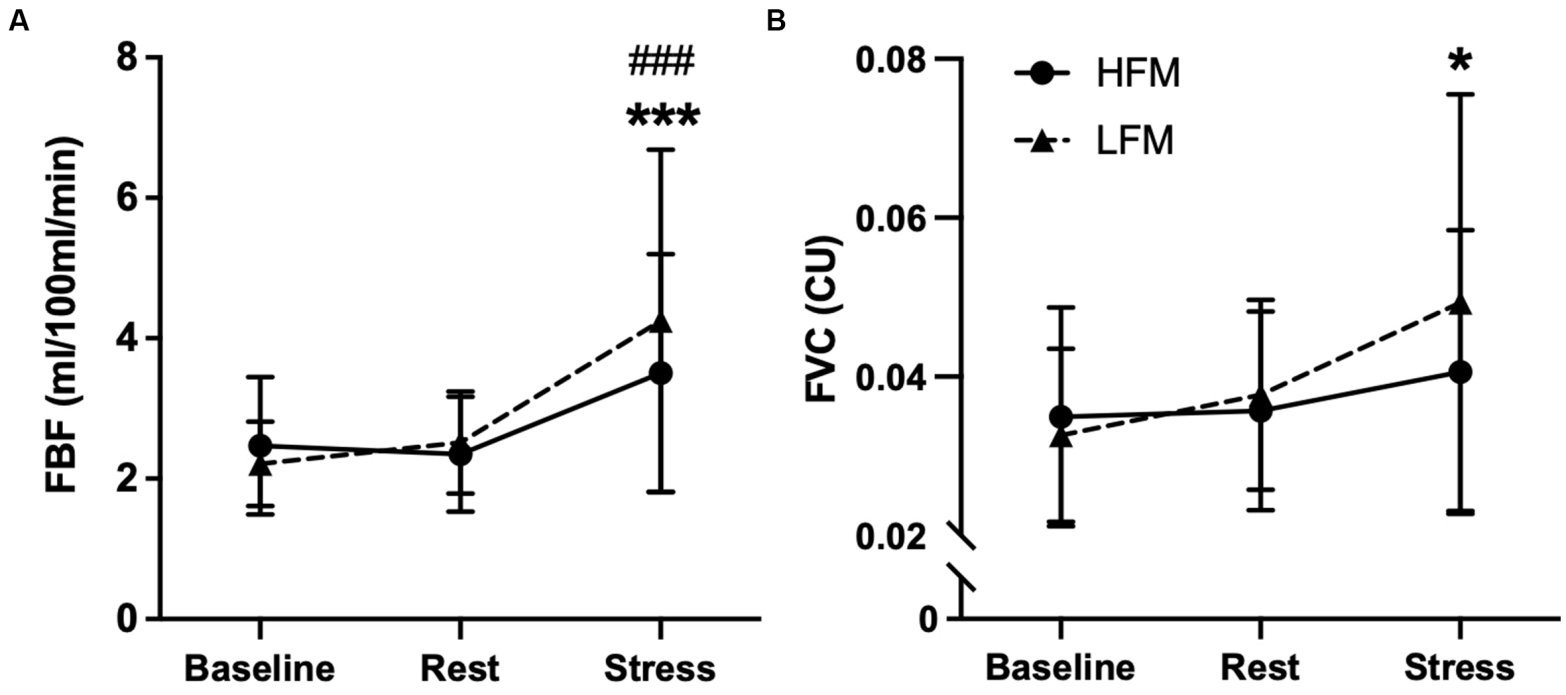
Time course of forearm blood flow [FBF **(A)** & FVC **(B)**] during baseline, rest and stress following either a high-fat or low-fat meal. *n* = 21**(A)**/19**(B)**. Data are presented as Mean ± SD. * Significantly different from baseline, ^#^ significantly different from rest. ***/^###^*p* < 0.001, *p <0.05. FBF, forearm blood flow; FVC, forearm vascular conductance; HFM, high-fat meal; LFM, low-fat meal.

A 2 condition × 3 time ANOVA revealed an overall time effect for FBF (*n* = 21, *p* < 0.001) and FVC (*n* = 19, *p* = 0.007) (Figure 5). *Post hoc* analyses revealed that FBF was significantly higher during stress compared to both baseline (*p* < 0.001) and rest (*p* < 0.001). Similarly, FVC was significantly higher during stress compared to baseline (*p* = 0.023) but not rest (*p* = 0.062). There were no condition nor condition × time interaction effects for FBF (condition: *p* = 0.357, interaction: *p* = 0.136) or FVC (condition: *p* = 0.432, interaction: *p* = 0.188).

### 3.7. Flow-mediated dilatation following mental stress

**Figure 6:**
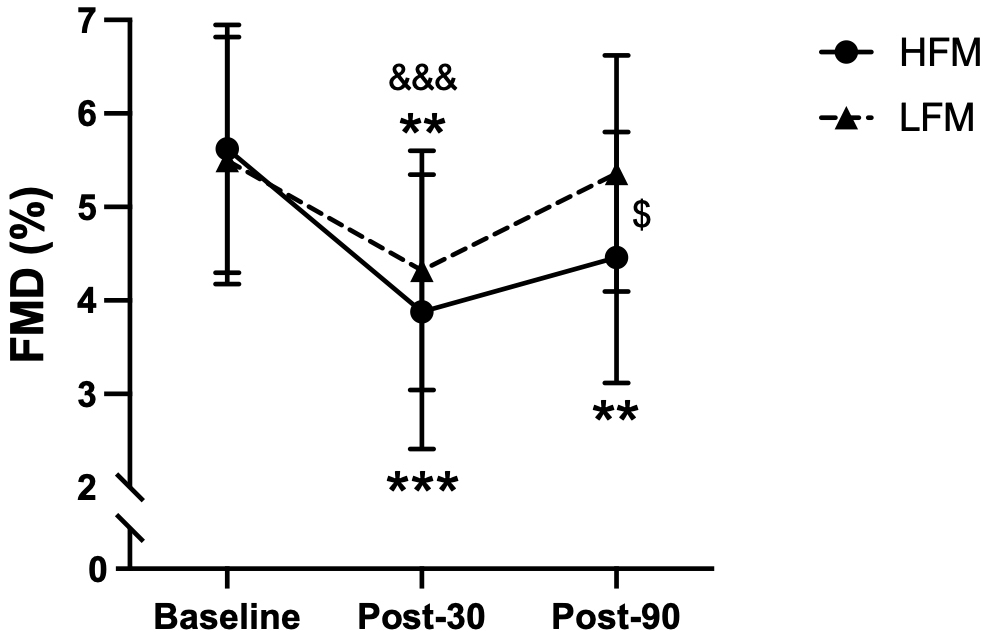
Time course of brachial artery FMD (%) during baseline, post-30 and post-90 following either a high-fat or low-fat meal. *n* = 21. Data are presented as Mean ± SD. ^$^ Significantly different between conditions, * significantly different compared to baseline, ^&^ significantly different compared to post-90. ***/^&&&^*p <* 0.001, **p < 0.01, ^$^*p* < 0.05. FMD, Flow-mediated dilatation; HFM, high-fat meal; LFM, low-fat meal.

Brachial FMD following mental stress is reported in Figure 6 (*n* = 21). A 2 condition × 3 time ANOVA revealed a significant time effect for brachial FMD (*p* < 0.001). Post-hoc analyses showed that FMD at 30 min post-stress was significantly lower compared to both baseline (*p* < 0.001) and 90 min post-stress (*p* = 0.001), and FMD at 90 min post-stress was lower compared to baseline (*p* = 0.048). Furthermore, there was a significant condition × time interaction effect for brachial FMD (*p* = 0.008) (Figure 6). Further exploration of this interaction effect revealed that FMD was significantly lower 90 min post-stress in the high-fat condition compared to the low-fat condition (*p* = 0.018). Examination of the time effects in both conditions separately, showed that in the high-fat condition, there was no significant difference in FMD between 30 min and 90 min post-stress (*p* = 0.134), but both were different from baseline (*p* < 0.001, *p* = 0.003, respectively). In the low-fat condition, FMD was significantly lower at 30 min post-stress compared to both baseline (*p* = 0.008) and 90 min post-stress (p < 0.001), but there was no difference between baseline and 90 min post-stress (*p* = 1.000). In other words, in the high-fat condition, FMD remained significantly lower up to 90 min post-stress, whereas in the low-fat condition, FMD was no longer significantly different from baseline 90 min post-stress. There was no significant condition effect for brachial FMD (*p* = 0.085).

Brachial arterial diameter (*n* = 21), positive blood flow (*n* = 21), and negative blood flow (*n* = 19) are reported in Table 5. There was no significant effect of condition (*p* = 0.123), time (*p* = 0.316) or condition × time interaction (*p* = 0.219) for arterial diameter, suggesting satisfactory sonography. There was no significant time (*p* = 0.749) or condition × time interaction (*p* = 0.107) effect for positive blood flow, yet a significant condition effect (*p* = 0.002), with a greater blood flow in the high-fat compared to the low-fat condition. There was no significant condition (*p* = 0.421) or condition × time interaction (*p* = 0.723) effect for negative blood flow, but a significant time effect (*p* < 0.001) with a significantly greater negative blood flow 30 min post-stress compared to baseline and 90 min post-stress.

**Table 5:** Mean ± SD brachial arterial diameter following mental stress (*n* = 21).

	High-fat meal			Low-fat meal		
Timepoint	Baseline	Post-30	Post-90	Baseline	Post-30	Post-90
Diameter (mm)	3.77 ± 0.63	3.83 ± 0.67	3.84 ± 0.67	3.76 ± 0.67	3.76 ± 0.69	3.75 ± 0.70
Positive blood flow (ml∙min^−^1)	98.80 ± 54.98	107.14 ± 61.39	105.30 ± 69.20	86.27 ± 58.49	70.82 ± 40.66	81.86 ± 51.18
Negative blood flow (ml∙min^−^1)	–7.84 ± 10.42	–17.04 ± 18.98	–11.51 ± 15.43	–10.90 ± 10.35	–17.74 ± 14.65	–13.10 ± 14.78

### 3.8. Sex differences

All analyses were also carried out with sex as a between-subject variable. There were no significant condition × sex interaction effects for FBF (*p* = 0.380), FVC (*p* = 0.952), HR (*p* = 0.665), HRV (*p* = 0.947), PEP (*p* = 0.856), SBP (*p* = 0.746), DBP (*p* = 0.826), FMD (*p* = 0.710) or TAG (*p* = 0.404). There were no significant time × sex interaction effects for FBF (*p* = 0.207), FVC (*p* = 0.444), HR (*p* = 0.612), HRV (*p* = 0.193), PEP (*p* = 0.846), DBP (*p* = 0.065), FMD (*p* = 0.893) or TAG (*p* = 0.799). However, there was a significant time × sex interaction for SBP (*p* = 0.008), whereby males have a significantly lower SBP compared to females at pre-intervention baseline (*p* = 0.015), and SBP significantly increases from pre-intervention baseline to rest for males (*p* = 0.035) but not for females (*p* = 1.000). Finally, there was no significant condition × time × sex interaction for FBF (*p* = 0.665), FVC (*p* = 0.930), HR (*p* = 0.180), HRV (*p* = 0.444), PEP (*p* = 0.186), SBP (*p* = 0.397), DBP (*p* = 0.170), FMD (*p* = 0.908) or TAG (*p* = 0.357).

## 4. Discussion

The current study investigated the effects of fat consumption and stress on the vasculature in young healthy adults. As expected, and shown previously, we observed peripheral vasodilation and cardiovascular perturbations during mental stress and a decline in brachial FMD 30 min following mental stress. To our knowledge, this is the first study to show that consumption of a high-fat meal prevents the recovery of endothelial function 90 min following mental stress. Fat consumption did not influence peripheral vasodilation or cardiovascular (HR, PEP, HRV, BP) responses during stress.

As previously shown, mental stress induced an acute increase in FBF (12, 37). Similarly, we observed a decline in brachial FMD 30 min following mental stress (1.74 and 1.18% decline following high and low-fat meals, respectively), in line with our (37) and other previous studies in healthy adults (42–46). NO has been implicated in both the peripheral vasodilation during mental stress and the decline in FMD following mental stress. Sympathetic activation and parasympathetic withdrawal are responsible for increased cardiovascular reactivity during stress (47), and this autonomic activity also contributes to NO-mediated vasodilation during stress (48, 49). Following stress, elevated levels of cortisol and inflammatory markers (42, 50) have been suggested to contribute to post-stress endothelial dysfunction (51) through a reduction in NO bioavailability (52, 53).

No change in resting or stress-induced FBF was reported following the high-fat meal in comparison to the low-fat meal control condition. Only a few studies have investigated the effect of a high-fat meal on resting FBF, with mixed findings reporting attenuated (54), improved (55) and no change (56) in peripheral vasodilation. However, differences in fat content, i.e., 30 g fat (54) vs. 60 g fat (56), calorie count, as well as other nutrients taken together with fat (55, 57) make a direct comparison between the studies challenging. Furthermore, the timing of the FBF assessment may also influence these results. For example, the present study measured resting FBF 1 h 15 min post-fat consumption, whilst Shimabukuro et al. (2007) measured FBF 2 h following a high-fat meal, and other studies’ assessments have been at least 3 h post-fat intake, which is more in line with the fat-induced peak in TAG (58–60). Therefore, future studies should investigate resting FBF for longer periods following fat consumption, to provide evidence of the mechanisms involved in peripheral vasodilation following fat consumption.

Stress-induced FBF after a high-fat meal was only investigated by one other study, which reported attenuated FBF responses to stress post-fat intake, in contrast with our results (even though we used a comparable intervention) (56). There are, however, other notable methodological differences between the studies, such as provocativeness of the task, order of meal conditions, and control condition (fasted versus low-fat in the current study). The task used in the current study induced similar and substantial HR responses in both conditions (27 bpm increase in low-fat condition, 28 bpm increase in high-fat condition) in line with previous studies that have used this protocol (12, 35, 36). The shorter task applied by Gowdak and colleagues (56) induced a lower HR response, which was significantly lower after the high-fat meal (6 bpm increase) compared to the fasted condition (10 bpm increase). In the present study, the order of conditions was counterbalanced between participants and the conditions were completed on separate days. In contrast, in the previous study, all participants completed the fasted condition prior to the high-fat condition, and both conditions were completed on the same day (56). Finally, the previous study compared a high-fat condition with a fasted condition, whereas the control condition in the current study was a low-fat meal, meaning both conditions were similar and postprandial with the exception being a difference in fat content. Therefore, it is difficult to determine if the reduced FBF reported by Gowdak and colleagues (56) following a high-fat meal is due to an order effect of testing, or just a postprandial effect, or fat intake itself.

Fat consumption did not influence the observed decline in FMD 30 min post-stress yet did impact the recovery of FMD 90 min post-stress. Some studies have shown a < 1% decrease in FMD following fat consumption (24, 61) and a more consistent 1–3% decline in FMD following stress (15), yet these findings suggest there is no additive (or interaction) effect of fat and stress on FMD 30 min post-stress. However, in the present study, fat consumption did impair the recovery of FMD at 90 min following stress, suggesting that consuming fat during stressful periods can prolong impairments in endothelial function in healthy young adults. On the other hand, Poitras et al. (32) reported no effect of fat consumption on FMD 10 min following stress, which is in agreement with our results, as earlier time points (30 min post-stress) do not seem to result in worsened endothelial function. However, direct comparisons between the two studies must be taken with caution as there are significant methodological differences. Poitras and colleagues (32), subjected participants to four consecutive provocative stress tasks (50 min apart, inducing 20 bpm increases in HR) and no effects on FMD were detected 10 min post stress, in both low and high-fat conditions, indicating no impact of stress alone on endothelial function. Indeed, the literature presents more consistent FMD impairments 30 to 90 min post-stress (which we targeted in the current study) (15), with one study showing no FMD impairment 15 min post-stress (62). Furthermore, it is well-established that the autonomic nervous system is stimulated during mental stress (47), and it is possible that sympathetic activation remains elevated at 10 min post-stress, making the FMD assessments less reliable. Importantly, the design of the present study allowed us to determine the impact of fat consumption on FMD recovery following stress without the confounder of an activated sympathetic nervous system, which has not been possible with previous study designs. Overall, our data suggests that reduced post-stress endothelial function after fat consumption is only apparent at least 90 min post stress, whilst at earlier time points (30 min) no fat-stress interaction is detected.

The mechanisms by which fat consumption delays the recovery of FMD following mental stress are not known. TAG and C-reactive protein (CRP) have been evidenced to be increased in circulation 2–4 h post-fat consumption (58–60), as supported in the present study. This is reflected in our FMD assessments and may explain why intake of fat slows down endothelial function’s recovery 90 min post-stress, but not 30 min post-stress (2 h post-fat intake). The mechanisms driving hypertriglyceridemia-induced endothelial dysfunction are not clear. However, there is evidence that triglyceride-rich lipoprotein particles may cause direct injury to the vascular wall (63). Alternatively, fat consumption may induce endothelial dysfunction indirectly by increasing oxidative stress, as hypertriglyceridemia has been evidenced to upregulate superoxide anion, a precursor of ROS (27). Finally, elevations in triglycerides and CRP following fat consumption have been shown to stimulate vasoconstrictor ET-1 and inflammatory markers (29). All of these mechanisms can subsequently reduce endothelium-derived NO (30), thus impairing endothelial function, and should be measured in future work. Whilst insulin and TAG start to increase in circulation 30 min following fat ingestion (58), they are unlikely to have reached their peak during our stress task and FBF assessment (1 h 15 min post-fat consumption), which may explain our null results for FBF. Furthermore, even though postprandial increases in TAG and insulin are likely to modulate FBF, the direction of this response is not well-established (55, 57). As FMD was our primary outcome, this informed the choice of timing post-fat consumption and, ensures that we are simultaneously targeting the timeframes in which circulatory TAGs rise and NO declines post-stress. Future studies should be designed to target the FBF response timeframe, allowing the direct assessment of the impact of fat on vascular responses during stress.

In line with previous research, the current study showed no influence of fat consumption on resting cardiovascular parameters (64, 65). Whilst fat consumption could influence sympathetic activation, there is evidence that other nutrients and consumption of food in general have a predominant role (66, 67). This is supported by the observed postprandial increase in HR at rest, following consumption of both high and low-fat meals. As expected, mental stress induced an immediate change in HR, BP, and measures of sympathetic and parasympathetic activity, which was not impacted by fat consumption. Perhaps this is unsurprising, as fat consumption does not impact resting cardiovascular parameters, so it is also unlikely to modify cardiovascular responses during stress. There is little evidence of the impact of fat on cardiovascular responses during stress, with vast methodological differences (56), and hence, future research is required in order to make a firm conclusion. Furthermore, while fat consumption does not seem to influence cardiovascular and vasodilatory responses during stress, fat intake may influence resting cardiovascular function following stress. Therefore, future research should similarly assess cardiovascular and vascular changes alongside FMD measurements following stress.

## 5. Limitations

One of the limitations in the present study is that the high-fat and low-fat meals were not tailored to individual metabolic rate. This is likely to translate into a higher variability in responses to fat-intake between participants, which can be considered more ecologically valid and further highlights the significance of our results. Furthermore, Jackson et al. (2007)‘s review (25) suggests that approximately 50 g fat is sufficient to impact endothelial function, which is comparable to the 56.5 g dose of fat in the present study, previously shown to impair endothelial function (24).

The sample used for this study was moderate, yet a robust crossover design was employed, and as effect sizes for non-significant findings are small (interaction effect sizes for FBF, HR, HRV, PEP, and BP were 0.11, 0.08, 0.04, 0.04, and 0.02, respectively), a lack of power was not likely to drive these results. Furthermore, post-hoc power analyses revealed that a sample of 21 participants, power at 90% and alpha set at 0.05, allowed the detection of a medium size interaction effect (0.33) for our primary outcome measure, brachial FMD (68).

The present study population is estimated to have a relatively healthier habitual diet compared to the UK population. For example, 62% of participants consumed at least 5 portions of fruit and vegetables (average 5.7 portions/day), compared to 28% of UK adults (average 3.7 portions/day) (69). Similarly, only 19% of participants exceeded the recommended saturated fat value compared to 75% of UK adults (70). Therefore, the present study sample may represent a healthier population, which highlights additional significance of our observations. It is highly likely that such fat-induced impairments in endothelial function may be further aggravated in a general population with a poorer habitual diet, and particularly in individuals at risk of CVD, such as obese or hypertensive, known to have disturbed vascular responses to stress (14). Therefore, future research should target these populations. Furthermore, it would be interesting to understand how aspects of baseline characteristics, such as diet, fitness level, blood pressure and TAG concentration might influence responses to stress following a high-fat meal, yet a larger sample is required to address this. Therefore, future research should explore what characteristics may put people at higher risk from consuming fat during stress.

## 6. Conclusion

This study demonstrates the detrimental impact of a high-fat meal and stress on endothelial function. Whilst fat had no effect on vascular and cardiovascular responses during stress, the prolonged impairment in endothelial function following stress is significant. Given that a 1% impairment in FMD has been correlated with a 13% increase in CVD risk (16), future work should investigate how long such fat and stress-induced impairments in endothelial function last. This might be particularly critical if the combination of stress and fat ingestion becomes chronic, preventing the endothelium’s chance to fully recover. Given the documented trend towards consumption of high-fat foods during periods of heightened stress, our data can have important implications for future dietary recommendations to protect the vascular system during periods of enhanced vulnerability (such as those rendered by stress).

## Data availability statement

The raw data supporting the conclusions of this article will be made available by the authors, without undue reservation.

## Ethics statement

The studies involving humans were approved by University of Birmingham Science, Technology, Engineering and Mathematics ethics committee. The studies were conducted in accordance with the local legislation and institutional requirements. The participants provided their written informed consent to participate in this study.

## Author contributions

RB: Conceptualization, Data curation, Formal analysis, Funding acquisition, Investigation, Methodology, Writing – original draft. SW: Data curation, Formal analysis, Investigation, Writing – review & editing. CR: Conceptualization, Data curation, Formal analysis, Funding acquisition, Investigation, Methodology, Project administration, Resources, Supervision, Writing – review & editing. JV: Conceptualization, Data curation, Formal analysis, Funding acquisition, Investigation, Methodology, Project administration, Resources, Supervision, Writing – review & editing.
